# Gaze direction and face orientation modulate perceptual sensitivity to faces under interocular suppression

**DOI:** 10.1038/s41598-022-11717-4

**Published:** 2022-05-10

**Authors:** Renzo C. Lanfranco, Timo Stein, Hugh Rabagliati, David Carmel

**Affiliations:** 1 Department of Psychology, University of Edinburgh, Edinburgh, UK; 2 Department of Neuroscience, Karolinska Institutet, Stockholm, Sweden; 3 Department of Psychology, University of Amsterdam, Amsterdam, The Netherlands; 4 School of Psychology, Victoria University of Wellington, Wellington, New Zealand

**Keywords:** Consciousness, Perception, Psychology

## Abstract

Faces convey information essential for social interaction. Their importance has prompted suggestions that some facial features may be processed unconsciously. Although some studies have provided empirical support for this idea, it remains unclear whether these findings were due to perceptual processing or to post-perceptual decisional factors. Evidence for unconscious processing of facial features has predominantly come from the Breaking Continuous Flash Suppression (b-CFS) paradigm, which measures the time it takes different stimuli to overcome interocular suppression. For example, previous studies have found that upright faces are reported faster than inverted faces, and direct-gaze faces are reported faster than averted-gaze faces. However, this procedure suffers from important problems: observers can decide how much information they receive before committing to a report, so their detection responses may be influenced by differences in decision criteria and by stimulus identification. Here, we developed a new procedure that uses predefined exposure durations, enabling independent measurement of perceptual sensitivity and decision criteria. We found higher detection sensitivity to both upright and direct-gaze (compared to inverted and averted-gaze) faces, with no effects on decisional factors. For identification, we found both greater sensitivity and more liberal criteria for upright faces. Our findings demonstrate that face orientation and gaze direction influence perceptual sensitivity, indicating that these facial features may be processed unconsciously.

## Introduction

Facial features provide essential information about others’ mental states and intentions, and are remarkably effective at capturing attention^1^ even from early infancy^2,3^. A number of reports have even claimed that some facial features can be processed unconsciously^4–10^, with the implication that faces might be special stimuli, whose processing is prioritised to the extent that it does not require awareness. However, concerns about these findings have been raised both in terms of their replicability and interpretation^11–19^. In particular, and as explained in detail below, even the findings that replicate may not in fact reflect detection sensitivity to facial features, but instead could reflect differences in the biases and criteria that participants use during face processing tasks. This latter concern is particularly acute because the most popular recent method used to study unconscious face processing, the Breaking Continuous Flash Suppression technique (b-CFS), is unable to distinguish sensitivity from criterion and response bias. Do the configural features of a face and its gaze direction affect how faces gain access to awareness or just post-perceptual factors such as decision criteria? Here, we address this issue by focusing on two specific claims about facial features—first, that upright faces reach awareness faster than inverted faces, and second, that faces with direct gaze reach awareness faster than faces with averted gaze.

We test these claims using a more comprehensive method, which replaces response times (RTs) with measures based on signal-detection theory; to do so, we combine interocular suppression with the psychophysical method of constant stimuli, avoiding the problems inherent in b-CFS and allowing us to assess how face orientation and gaze direction modulate perceptual sensitivity to faces initially suppressed from awareness. If face configuration and gaze direction affected perceptual sensitivity, this would indicate in our method that these facial features affect basic perception, perhaps mediated by unconscious processing. However, if face configuration and gaze direction influenced decisional criteria only, this would imply that these effects emerged at later processing stages, thus requiring conscious awareness.

A rich body of studies has claimed that facial features such as gaze direction^9,20^, emotional expression^10,21–23^, familiarity^7^, and attractiveness^24^ can be processed unconsciously. To render images invisible, these studies have employed Continuous Flash Suppression (CFS), a strong interocular suppression procedure^25^, in which a stimulus presented to one eye is suppressed from awareness by Mondrian-like masks flashed to the other eye. In the b-CFS variant, participants are asked to provide a response as soon as the invisible stimulus breaks through suppression into awareness^26^, with the assumption that stimuli which are processed with higher priority will break through into awareness faster^27^. Previous work using this procedure has found that faces break through suppression faster when shown in upright orientation than in inverted orientation^8^, when expressing fear compared to a neutral expression^10^, or when making eye contact compared to looking away^9^.

Although the b-CFS paradigm has been widely used to provide evidence for differential access of visual features to awareness, its reliance on RTs raises some concerns. Importantly, RTs are a measure of overall processing speed, encompassing the many processes that go into producing a *speeded* (not just correct) response. RTs are not an isolated measurement of perceptual sensitivity, and thus using them precludes conclusions that make specific claims about perceptual sensitivity to suppressed stimuli. A crucial concern is that differences in detection times could reflect differences in decision criteria rather than differences in perceptual sensitivity. When suppressed stimuli break into awareness they often do so gradually, which means that participants have to make a decision as to whether—and when—to report a partially-perceived stimulus. Their criteria for making these decisions may vary by stimulus category. For example, even if perceptual sensitivity for upright and inverted faces was identical, upright faces might be reported faster simply because they are associated with a more liberal criterion for the decision to press a key, perhaps because they look more familiar, resulting in greater confidence. Similarly, even if perceptual sensitivity for direct-gaze and averted-gaze faces was identical, direct-gaze faces might be reported faster simply because they are associated with a more liberal criterion for the decision to press a key, perhaps due to their personal social relevance rather than because they are more visible. Alternatively, participants may be inclined to visually explore a certain stimulus category more exhaustively than another before deciding to commit to a response, thus leading to a more conservative criterion and thereby to a slower response. The implication of this is that differences in breakthrough times may not be due to differential sensitivity to stimulus categories but rather to differential decision criteria (i.e. the willingness to report a signal). This potential confounding effect of decision criteria could have major theoretical implications—if the face-inversion effect and/or the eye-contact effect are due to differences in decision criteria rather than perceptual sensitivity, it would suggest that social cognitive processes that rely on face processing may require some degree of conscious awareness to unfold. While differences in decision criteria may inform about implicit preferences or expectations, only differences in perceptual sensitivity can tell us about the ability of different stimulus categories to overcome suppression from awareness.

We are not the first to note that criterion issues are a concern in b-CFS studies, and indeed some b-CFS studies have tried to control for this problem. For instance, some researchers have included a non-rivalrous control condition (where the target stimuli are shown binocularly or monocularly on top of the flashing CFS masks) with the assumption that post-perceptual effects, such as differences in decision criteria, should have similar effects on suppressed and visible stimuli^8,19,28–34^. The underlying reasoning is that if a non-rivalrous condition emulates all processes that are not CFS-specific but contribute to differences in RTs, any larger differences between stimulus categories found in the rivalrous b-CFS condition (compared to the visible control condition) should index unconscious processing differences. However, non-rivalrous conditions do not effectively control for decision criteria. For example, targets in non-rivalrous control conditions are more easily discernible from the mask^35^, meaning there is less uncertainty about them; and the level of uncertainty is known to affect decision criteria^36^ and may do so differentially for different stimulus categories. Visible conditions therefore differ in a substantive way from CFS conditions, meaning they are not valid controls.

Another proposed method for controlling for differences in decision criteria is to ask participants to perform an orthogonal task, such as reporting a stimulus feature that is irrelevant to the experimental manipulation (e.g. Gayet et al.^37^; Salomon et al.^38^). This approach assumes that if participants do not need to identify or make decisions about the experimentally critical but task-irrelevant feature, their RTs will reflect processing that is unaffected by differences in identification performance or decision criteria. However, this assumption is unjustified: Participants may still perceive (and thus make decisions about) the task-irrelevant feature, and their choice of how long to accumulate information on each trial may still be affected by their internal criterion for responding to that feature, or their ability to identify it, irrespective of its relevance for the task. Crucially, we cannot tell what factors will affect participants’ decision in any paradigm where they can freely choose how much perceptual evidence to gather (i.e. how long to look at the stimulus in a trial) before responding.

To assess perceptual sensitivity independently of decision criterion and dissociate detection from identification, we must use a method that does not rely on RTs (a measure of participants’ willingness to commit to a response), but rather on measures collected under conditions where perceptual evidence (e.g. exposure duration in a trial) is controlled by the experimenter. Here, we developed and tested a method that combines CFS with the method of constant stimuli, and thus does not suffer from the above problems. We used this method to test two well-established b-CFS findings that have been successfully replicated: the face-inversion effect and the eye-contact effect.

Even without suppression, upright faces are easier to recognise than inverted faces^39–43^. In line with this, the first published b-CFS study found that upright faces overcome suppression faster than inverted faces^8^. This face-inversion effect has been repeatedly replicated with b-CFS procedures^9,14,28,44,45^ and has been interpreted as evidence of unconscious holistic face processing. Similarly, faces that make eye contact appear to be processed in a special way. Without suppression, for example, eye contact draws attention towards the face, whereas averted gaze draws attention towards the gaze’s direction^46–49^. Multiple studies have shown that eye contact also promotes social learning from a very young age^50–53^. Using b-CFS, Stein et al.^9^ reported that suppressed human faces with direct gaze were detected faster than faces with averted gaze, suggesting a processing advantage driven by eye contact (the same study also replicated the aforementioned face-inversion effect). Subsequently, a number of other studies have supported the idea that direct gaze faces are (unconsciously) prioritised either by measuring breakthrough times directly^28,54,55^ or by measuring neural markers before the faces overcome suppression^20,31,56^.

In some of these studies, the task—to report stimulus location (on the left or right side of the screen)—was orthogonal to the hypothesis-relevant stimulus category (e.g. direct/averted gaze; Chen and Yeh^20^; Stein et al.^9^). However, as detailed above, shorter breakthrough times to direct-gaze faces do not necessarily reflect higher sensitivity, but could instead be due to a more liberal decision criterion: observers may simply require less evidence (and thus less time) for deciding to report that they have seen a face when its gaze is direct rather than averted. Thus, it is still unclear whether upright and direct-gaze faces break suppression faster. To ascertain this, it is necessary to demonstrate greater perceptual sensitivity to CFS-suppressed upright (compared to inverted) and direct-gaze (compared to averted) faces, under conditions that limit the influence of criteria over participants’ decisions.

To accomplish this, we presented CFS-suppressed stimuli for a range of predefined durations. On each trial, participants saw a face with direct or averted gaze that was presented in upright or inverted orientation. Following each display, participants reported the face’s location (left or right of fixation) and its identity (direct or averted gaze), as accurately as possible, with no speed pressure. We used signal detection analyses to establish how stimulus duration and type affected sensitivity and decision criteria for both of these reports. A similar stimulus-presentation approach was employed by Stein et al.^35^ (Experiment 3), who used four predetermined exposure durations and found that participants showed higher accuracy in reporting the location of upright versus inverted faces at all durations. Notably, however, they only measured accuracy; they did not use signal-detection measures to directly assess perceptual sensitivity. Furthermore, they did not account for identification processes that might affect accuracy, or for criterion differences in such identification processes.

First, in Experiment 1, we verified the robustness of previous b-CFS findings and the suitability of our stimuli and setup, by conducting a direct replication of Stein et al.’s^9^ second experiment, a b-CFS study that demonstrated faster RTs to upright than to inverted faces, and was the first to demonstrate faster responses to direct than to averted gaze faces. In Experiment 2 (pre-registered at https://aspredicted.org/qj4wf.pdf), we used our new method to acquire signal-detection measures for both face location (left/right side of the screen) and identification (direct/averted gaze) at each of seven exposure durations, ranging from 500 to 5695 ms. If face orientation and gaze direction modulate perceptual sensitivity under suppression, as suggested by previous b-CFS findings, we should find greater sensitivity for direct-gaze versus averted-gaze faces and for upright versus inverted faces. Data and materials are publicly available on the Open Science Framework (https://osf.io/uepgt/).

## Experiment 1

Experiment 1 was an exact replication of Experiment 2 reported by Stein et al.^9^, testing whether upright faces break through suppression faster than inverted faces, whether faces making eye contact break through suppression faster than averted gaze faces, and whether the factors of face orientation and gaze direction interact. We used the same Matlab scripts and stimuli as the original study but employed a larger sample (32 instead of 14 participants). The original study found a processing advantage for faces making eye contact. Additionally, upright faces broke through suppression faster than inverted faces. There was no interaction between these two effects.

### Methods

#### Participants

Thirty-two University of Edinburgh students (21 female; 4 left-handed; mean age 23.8, SD_age_ = 4.1) provided informed consent and were paid £3 for participation. All had normal or corrected-to-normal vision and reported no history of neurological or psychiatric disorders. Both experiments reported here were approved by the University of Edinburgh Psychology Research Ethics Committee. All participants provided informed consent in accordance with the Declaration of Helsinki.

Originally, Stein et al.^9^ employed only 14 participants in each of their experiments. Because concerns have been expressed regarding power limitations in psychophysical studies^57,58^, we more than doubled the number of participants to 32. We note that our sample size, which was ~ 2.3 times larger than the original, provided 99% power to detect an effect of size ${\eta {\mathrm{p}}}{2}=0.4$, which corresponds to the effect size reported in another replication of Stein et al.’s^9^ experiment by Akechi et al.^28^; although publication bias and other factors may inflate effect sizes in the published record, a power estimate of 0.99 indicates that our sample size provided sufficient power to detect even a much smaller effect.

**Figure 1:**
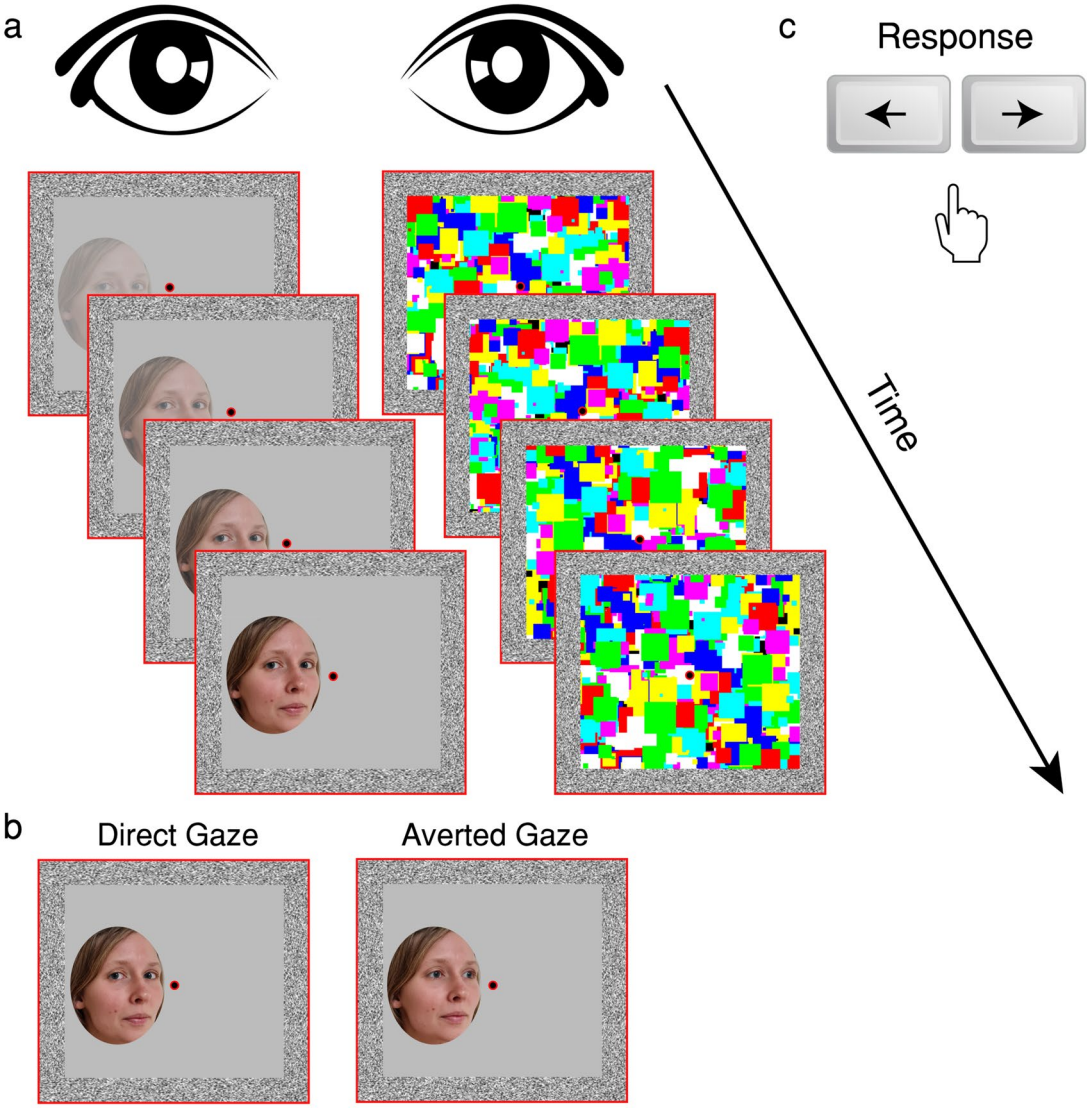
Schematic description of a trial in Experiment 1 (replication study). (**a**) The mask stimuli were shown at 100% contrast whereas the target stimulus increased in contrast linearly from 0 to 100% over 1 s. (**b**) Example of direct-gaze and averted-gaze faces. (**c**) The trial ended when the participant gave a response (left or right) or after 10 s.

For copyright reasons, the illustrative faces shown in Figs. 1 and 3 were not among those used in either experiment. The model in these figures provided informed consent and permission to publish her face images and did not participate in either experiment. The stimuli used in the experiments and datasets analysed can be found in the Open Science Framework (OSF) repository: https://osf.io/uepgt/.

#### Stimuli

In both experiments reported here, stimuli were presented on a 19-inch CRT monitor in a dimly lit room. The monitor was connected to a computer running Matlab 2014a (Mathworks, Inc) using the Cogent 2000 toolbox (http://www.vislab.ucl.ac.uk/cogent.php). A chin rest and mirror stereoscope were positioned 57 cm from the monitor, with a vertical divider splitting the display so each eye only saw half of the screen.

Figure 1a illustrates the display and stimuli. Two red frames containing binocular alignment contours (random noise pixels around the inside border of the frame; squares measuring 10.6° × 10.6°, width 0.8° × 0.8°) appeared side by side on the screen, supporting binocular alignment through the mirror stereoscope such that only a single frame was perceived. A red fixation dot (0.7° × 0.7°) was presented in the centre of each frame. Rectangular multicoloured Mondrian-like masks differing in size, rotation, and position were flashed at 10 Hz to one eye while a face stimulus was presented to the other eye.

We employed the same twelve face stimuli used by Stein et al.^9^ and other b-CFS studies^28,31,55^; these face stimuli were previously used^49,59–61^ and perceived gaze direction was validated^61^ in earlier non-CFS gaze direction studies. In these images, the face is laterally averted either to the left or to the right, and the eyes are also averted to either the left or right, giving the impression of either averted or direct gaze, depending on whether gaze direction matches head direction. For instance, from the viewer’s perspective, in the case of faces averted to the right, eyes directed to the left were classified as direct gaze and eyes directed to the right were classified as averted gaze, which ensures that eye symmetry is the same in direct-gaze faces and indirect-gaze faces (see Senju and Hasegawa^49^, for details of stimulus creation). Stimuli were cropped to oval shapes (3.3° × 4.6°), equalised for contrast and luminance and the edges were blurred into the grey background. Inverted faces were created by turning upright faces 180°.

#### Procedure

Participants were instructed to focus on the fixation dot with both eyes open, avoid blinking as much as possible, and not look elsewhere.

The procedure on each trial is shown in Fig. 1a. The red frames and binocular alignment contours were continuously present during the experiment. At the start of each trial, fixation dots were presented binocularly for 1 s. Then, one eye was shown the CFS mask—Mondrian-like patterns changing at 10 Hz—and a face was introduced to the other eye. The face’s contrast ramped up linearly from 0 to 100% over 1 s and then remained constant until either the participant responded, or 10 s passed, at which point the face, fixation dots, and mask disappeared during a 1.5 s intertrial interval (ITI). The eye receiving the mask was the same throughout the study but varied randomly between participants.

Face stimuli were presented either to the left or to the right of the fixation dot (horizontal fixation-to-centre distance 2.7°; Fig. 1b) at a random vertical position (maximum centre-to-horizontal-midline distance 2.1°). Participants were instructed to press the left or right arrow key on the keyboard to indicate the location of the face as soon as they became aware of its presence (Fig. 1c).

The experiment consisted of 192 randomly ordered trials, which were evenly distributed over the two crossed experimental factors (gaze direction and face inversion), with the face appearing on each side of the visual field on half of the trials. A 5-min break was given halfway through the experiment. There were no practice trials. Half of the participants viewed a version of the faces with the head averted to the left and the other half viewed a version of the faces with the head averted to the right. The full experiment took around 20 min to complete.

#### Analysis and results

We calculated mean RTs based on trials with correct responses (98.8% of all trials). Trials with no response were treated as missing data (< 5% for each participant). A preliminary mixed analysis of variance (ANOVA) on mean RTs, which included the factors of gaze direction (direct or averted) and face orientation (upright or inverted) as within-subject factors, and head direction (left or right) as a between-subjects factor, showed no main effect of head direction nor any interaction of this factor with any other factor (all relevant p-values > 0.1), so this factor was collapsed in further analyses.

**Figure 2:**
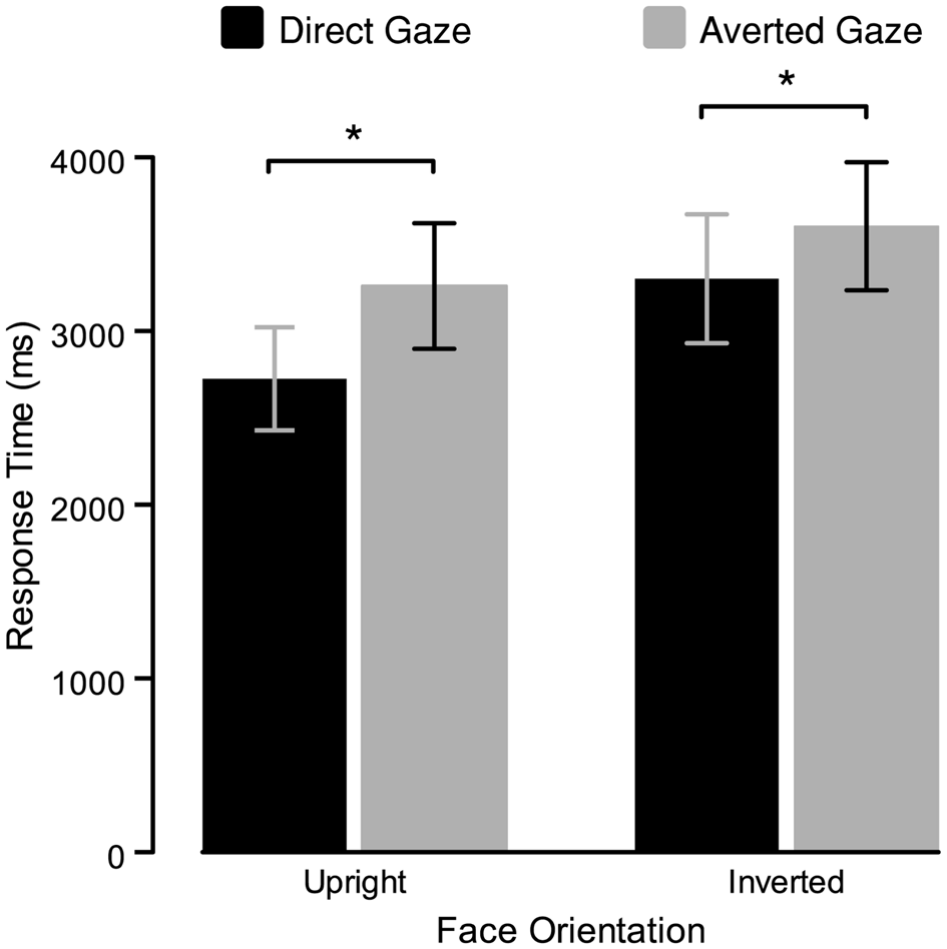
Results of Experiment 1. Bars indicate mean RTs for detection of CFS-masked faces. Asterisks index statistically significant differences. Error bars represent 95% confidence intervals (CI).

To examine whether upright and direct-gaze faces elicit faster breakthrough reports than inverted and averted-gaze faces, as Stein et al.^9^ found, we entered RTs into a 2 (gaze direction: direct, averted) × 2 (orientation: upright, inverted) repeated-measures ANOVA (Fig. 2). Critically, there was a main effect of gaze direction, with faster RTs for direct-gaze faces (*M* = 3016.8 [*SD* = 962.9]) than for averted-gaze faces (*M* = 3436 [1020.3]), $\left(F{(1, 31)}=54.14,p<.001,\eta{\mathrm{ p}}{2}=0.636\right).$ There was also a main effect of orientation, with faster RTs for upright faces (*M* = 2996.1 [950.5]) than for inverted faces (*M* = 3456.8 [1030.9]), $\left(F{(1, 31)}=75.72,p<.001,\eta{\mathrm{ p}}{2}=0.710\right)$. Finally, and similar to Stein et al.^9^, although the difference between direct and averted gaze was numerically larger for upright (*M*_difference_ = 535 ms [900]) than for inverted faces (*M*_difference_ = 303.4 ms [1008.8]), and each of these simple effects was significant $\left({t}_{upright}\left(61.5\right)= -6.35, p<.001, d=-1.122; {t}_{inverted}\left(61.5\right)=-3.59, p=.004, d=-0.635\right)$, they did not differ significantly from each other, as indicated by the finding that the interaction between gaze direction and face orientation did not reach significance $\left(F{(1, 31)}=3.49, p=.071, \mathrm{\eta p}{2}=0.101\right)$. These results replicate all aspects of Stein et al.’s findings^9^.

We further examined the non-significant interaction with a Bayes factor analysis, using JASP^62^ (version 0.12.2), in which we ran a Bayesian repeated-measures ANOVA with a standard r-scale prior of width 0.5 (for fixed effects), with a Cauchy prior scale parameter for covariates of 0.354 (this default prior was used in all subsequent Bayes factor analyses). This provided a value of $BF{01}=1.338$ for the interaction, indicating that given the data, the null is only slightly more likely than the alternative hypothesis model (anecdotal evidence). Thus, these data are not strongly informative as to whether or not the eye-contact effect is smaller for inverted faces.

### Discussion

Experiment 1 replicated Stein et al.’s^9^ findings: direct-gaze faces broke through CFS faster than averted-gaze faces (eye-contact effect), and upright faces broke suppression faster than inverted faces (face-inversion effect; see also^6–8,34,35,63–65^. As in the original study, we did not find a significant interaction between these effects, which may have implications for the possible mechanisms underlying the eye-contact effect in b-CFS; we return to this issue in the General Discussion. However, while faster breakthrough times have previously been interpreted as suggesting prioritised unconscious processing, such findings do not rule out the potential influence of differential criteria. Therefore, we next examined whether eye contact and face inversion affect perceptual sensitivity when the duration of exposure to the stimulus is controlled.

## Experiment 2

To measure perceptual sensitivity independently of decision criteria, we used the same stimuli as in the b-CFS paradigm but presented them, on each trial, for one of seven fixed durations. After each stimulus presentation, participants judged both where on the screen the masked stimulus was shown (left or right; location task), and what that stimulus was (direct or averted gaze; identification task), with no speed pressure. We used signal detection analyses to assess sensitivity to both stimulus location and stimulus identity, as well as bias/criterion measures for making these judgments.

### Methods

#### Participants

Thirty-two University of Edinburgh students who had not participated in Experiment 1 provided informed consent and were paid £14 for participation. All had normal or corrected-to-normal vision and reported no history of neurological or psychiatric disorders. Three participants were excluded from analysis (see the “Analysis” section below); the remaining 29 participants (23 female; 3 left-handed) had a mean age of 24.6 (SD_age_ = 3.6). As in Experiment 1, our sample size of 29 provided > 99% power to detect an effect of size $\mathrm{\eta p}{2}=0.4$, which corresponds to the effect size reported in another replication of Stein et al.’s^9^ experiment by Akechi et al.^28^.

#### Stimuli and apparatus

Face stimuli in this experiment were the same as in Experiment 1. The visual display differed slightly from that of Experiment 1: instead of red frames and a red fixation dot, binocular vergence was maintained by two vertical vergence bars (width 1°, height 8°) that appeared to the left and right of stimuli in each eye from fixation (horizontal fixation-to-bar distance 3.1°), and a black fixation cross (0.7° × 0.7°).

#### Procedure

Participants were instructed to focus on the fixation cross with both eyes open and to avoid blinking during the trials.

**Figure 3:**
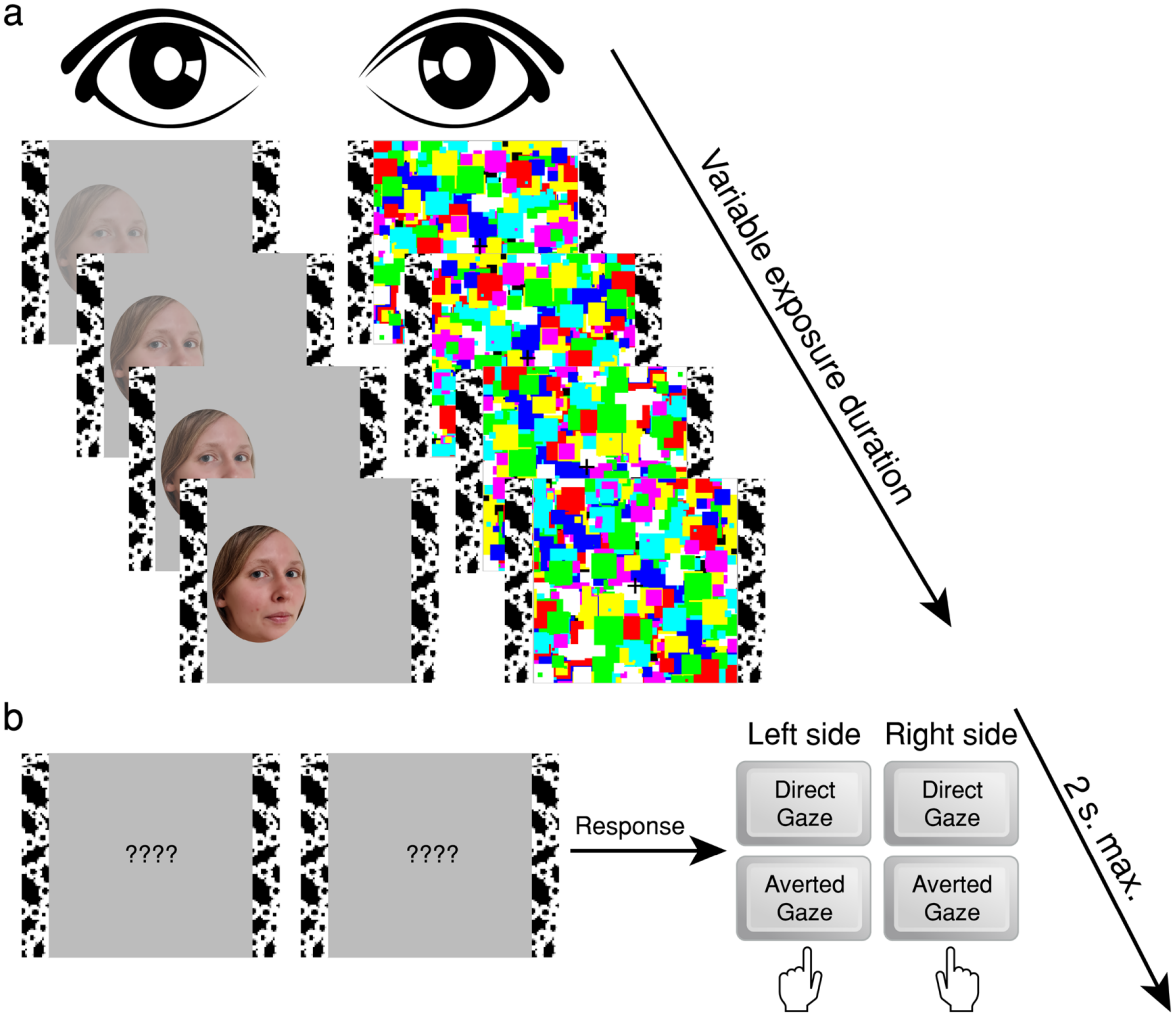
Schematic description of a trial in Experiment 2. (**a**) Stimulus presentation. Stimuli were presented for one of seven possible durations (500–5695 ms, equally spaced on a log scale). The contrast of the target image increased linearly from zero to 100% over the first second and then remained unchanged until the end of the trial. (**b**) Detection/identification response. Immediately following stimulus offset, a response cue was presented binocularly. Participants provided a single response to indicate both on which side of fixation the face had been shown and whether its gaze was direct or averted.

Two textured bars were presented to each eye continuously, to maintain stable vergence. Each trial began with a fixation cross presented binocularly between the textured bars (Fig. 3a). 200 ms later, the CFS mask (Mondrian-like patterns changing at 10 Hz) was presented to one eye, and a face image was introduced to the other eye, ramping up from 0 to 100% contrast over a 1-s period. On trials in which stimulus presentation was shorter than 1 s (see below), termination of presentation curtailed the change in contrast. On longer trials, face contrast remained at 100% until the end of the trial. The mask’s contrast was stable across the trial. Stimuli were presented for one of seven predefined durations, spaced equally on a log scale (500; 750; 1125; 1688; 2531; 3797; 5695 ms). This range of exposure durations was determined in piloting sessions that used exposures of 300–6000 ms; importantly, it encompasses the entire range of the mean breakthrough times found in Experiment 1 (all RTs ~ 2700–3700 ms).

After stimulus offset, the fixation cross was replaced with a response cue consisting of four question marks. Participants then had 2 s to respond. They were instructed to be as accurate as possible, but to make sure not to take longer than the 2-s response window (this is a non-speeded response because the instructions emphasised accuracy over speed, and the response window is substantially longer than typical RTs in detection and discrimination tasks for visible stimuli, which tend to be under 1 s^66^). Participants responded by pressing one of four keys (two on the left: left control and left shift; and two on the right: down arrow and up arrow) to indicate both where the face had been shown (left or right) and whether the face’s gaze was directed at them or averted (the keys for direct/averted gaze were counterbalanced; Fig. 3b). This single response thus provides measures of both detection (stimulus location), and identification (stimulus gaze). Following this response, a screen showing only the vergence bars was presented for an ITI of 1000 ms before the next trial began.

The experiment consisted of 1120 trials. Face orientation was blocked in a counterbalanced ABBABAAB BAABABBA order (70 trials/block, with A and B denoting upright and inverted faces, respectively, for half of the participants, or vice versa for the other half; face orientation was blocked to avoid any need to make a judgment of this factor on each trial as a preliminary step for gaze direction identification). Participants were given self-terminated breaks after every block and a compulsory 15-min break halfway through the experiment. Unlike Experiment 1, in Experiment 2 all participants viewed faces with the head averted both left and right, in order to maximise variation in the stimuli. For each face orientation, all combinations of face side (left/right), gaze (direct/averted), head direction (left/right), and stimulus duration (seven possible durations) were presented equally often in randomised order.

#### Analysis

We excluded data from three participants, in line with our pre-registered exclusion criteria: one failed to provide responses on more than 10% of trials and the other two did not show any increased accuracy as exposure duration increased, suggesting that they failed to attend to the task. For the remaining participants, trials that received no response (1.7% in total; < 5% per participant) were treated as missing data.

We used Signal Detection Theoretic (SDT) measures to assess how perceptual sensitivity and decision criteria changed across display durations. All measures were calculated for each combination of duration, face orientation, and gaze direction. To determine bias-independent sensitivity to face location (left or right; henceforth referred to as location d’), hits were defined as trials in which a face was displayed on the right and reported as being on the right, and false alarms (FAs) as trials in which a face was displayed on the left but reported as being on the right. To determine sensitivity to the presence of a face making eye contact (gaze direction identification d’), hits were defined as trials in which a direct-gaze face was shown and reported, and FAs as trials in which an averted-gaze face was shown but a direct-gaze face was reported (note that for the sake of simplicity, we refer to this measure as gaze direction identification d’; this denotes identification in the limited sense of identifying a specific detail within the stimulus—whether the gaze is direct or averted—rather than identifying who the person is). For each measure, we calculated d’ by subtracting the Z-transformed FA rate from the Z-transformed hit rate. Because in SDT terms the location task is a 2-alternative forced-choice task (requiring a decision on which of two sources of information contains the signal), for this task we divided d’ by the square root of 2^67,68^. We also calculated criterion measures ($C$) for both tasks, by multiplying each task’s sum of Z-transformed hit and FA rates by −0.5^68^. For the location task, this measure estimates each participant’s bias to respond left or right (henceforth referred to as response bias), with more positive values indicating a greater bias to respond “left”; however, as the direction of biases may vary across participants (and cancel out in averaging), we converted response bias scores to absolute values to assess the magnitude of response biases, independently of their direction. For the identification task, lower values indicate that the participant is more willing to report direct gaze. d’ and c values were entered into repeated-measures ANOVAs; Greenhouse–Geisser adjusted degrees of freedom were used when Mauchly’s test indicated a violation of the sphericity assumption.

### Results

#### Location sensitivity

Individual participants’ by-condition location d’ scores were entered into a preliminary repeated-measures ANOVA, which included gaze direction (direct or averted), face orientation (upright or inverted), head direction (left or right), and exposure duration. We found no main effect of head direction $\left(F{(1, 28)}=0.123, p=.731,\eta{\mathrm{ p}}{2}=0.005\right)$ nor any interaction of this factor with any other factor (all p-values > 0.1). Therefore, d’ scores in all further analyses were collapsed across head direction conditions.

**Figure 4:**
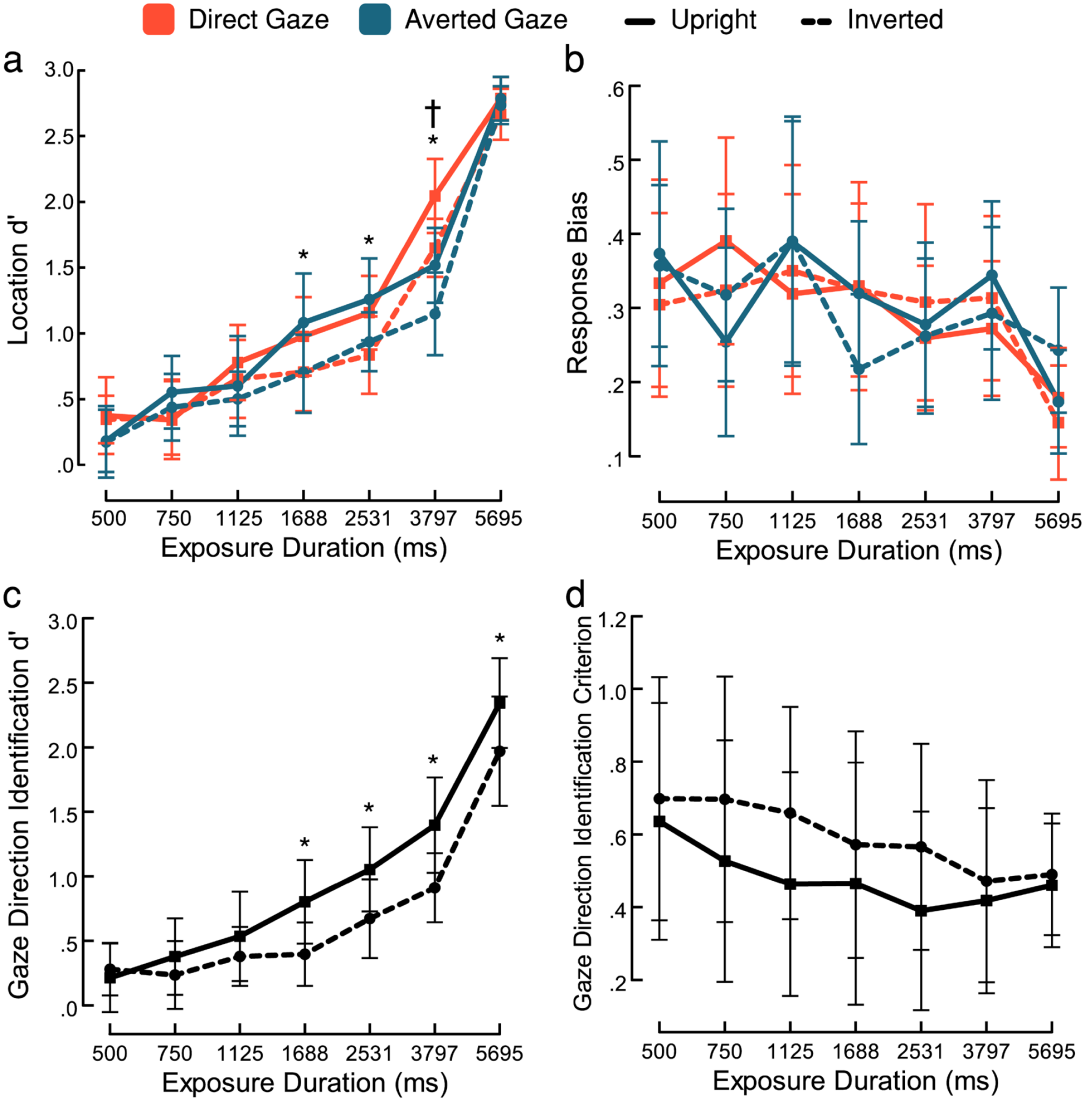
Results of Experiment 2. (**a**) Location sensitivity: d’ increased with exposure duration. A significant advantage for direct-gaze faces over averted-gaze faces is present at 3797 ms of exposure. A significant advantage for upright faces over inverted faces is present at 1688, 2531, and 3797 ms of exposure. (**b**) Absolute-value response bias scores for reporting location: bias decreased as exposure duration increased, but there was no difference in amount of response bias between gaze and orientation categories. (**c**) Identification sensitivity for gaze direction: identification d’ increased with exposure duration, and d’ was significantly greater for upright faces than inverted faces from 1688 ms of exposure. (**d**) Criterion scores for reporting direct gaze: upright faces exhibit a significantly more liberal criterion than inverted faces. Asterisks index statistically significant differences between face orientations. Daggers indicate statistically significant differences between gaze directions. Error bars represent 95% CI.

To examine how the manipulated factors affected face detection, we entered location d’ scores into a 2 (gaze direction: direct, averted) × 2 (face orientation: upright, inverted) × 7 (exposure durations) repeated-measures ANOVA. Unsurprisingly, there was a main effect of exposure duration (Fig. 4a), whereby sensitivity increased with exposure duration $\left(F{(2.6, 72.89)}=167.837,p<.001,\eta{\mathrm{ p}}{2}=0.857\right)$. Importantly, there was a main effect of gaze direction $\left(F{(1, 28)}=9.596,p=.004,\eta{\mathrm{ p}}{2}=0.255\right)$, confirming that participants were more sensitive to the location of direct-gaze faces $(\mathrm{M}=1.12 \left[1.07\right])$ than averted-gaze faces $(\mathrm{M}=1.04 \left[1.07\right])$. There was also a main effect of face orientation $\left(F{(1, 28)}=13.597,p<.001,\eta{\mathrm{ p}}{2}=0.327\right)$, indicating a sensitivity advantage for upright faces $(\mathrm{M}=1.17 \left[1.1\right])$ over inverted faces $(\mathrm{M}=0.99 \left[1.02\right])$*.* These main effects of gaze direction and orientation are consistent with the results of Experiment 1.

There was also a significant interaction between gaze direction and exposure duration $\left(F{(4.7, 131.65)}=12.24,p<.001,\eta{\mathrm{ p}}{2}=0.304\right)$*.* Bonferroni-corrected pairwise comparisons revealed that the advantage for direct-gaze faces over averted-gaze faces reached statistical significance at an exposure duration of 3797 ms $\left(t\left(196\right)= 7.82, p<.001, d=1.453\right)$. The interaction between face orientation and exposure duration was also significant $\left(F{(4.52, 126.56)}=4.096,p=.003,\eta{\mathrm{ p}}{2}=0.128\right)$*.* Bonferroni-corrected pairwise comparisons revealed that the advantage of upright faces over inverted faces was driven by significant differences at 1688 $\left(t\left(149\right)= 3.787, p=.02, d=0.703\right)$, 2531 $\left(t\left(149\right)= 3.751, p=.023, d=0.697\right)$ and at 3797 ms $\left(t\left(149\right)= 4.455, p<.001, d=0.827\right)$ of exposure (the advantage of upright over inverted faces is also evident, to a lesser extent, at 750 ms and 1125 ms; although these differences do not reach statistical significance at the very conservative threshold imposed by applying Bonferroni correction across seven durations, they affirm the overall pattern indicated by the main effect of orientation). Consistent with the findings of Stein et al.^9^ there was no interaction between gaze direction and face orientation $\left(F{(1, 28)}=0.098, p=.756,\eta{\mathrm{ p}}{2}=0.003\right)$, and no further three-way interaction with exposure duration $\left(F{(4.75, 132.9)}=0.368, p=.861,\eta{\mathrm{ p}}{2}=0.013\right)$.

The non-significant interaction between gaze direction and face orientation is similar to that found in Experiment 1, but similarly, it does not mean that the null hypothesis is necessarily true. By running a Bayesian repeated-measures ANOVA, we calculated a Bayes factor to test whether the present experiment’s data support the absence (null hypothesis model) of an interaction between gaze direction and face orientation. The Bayes factor indicated substantial evidence in favour of the null hypothesis model $\left(BF{01}=8.575\right)$. In other words, the results suggest that the location sensitivity advantage for direct-gaze faces did not depend on the orientation of the face.

These results, obtained with our new method, confirm the pattern of findings obtained using b-CFS in Experiment 1 and by Stein et al.^9^: Direct-gaze faces enjoyed a detection advantage over averted-gaze faces, and upright faces enjoyed an advantage over inverted faces. The absence of a significant interaction between gaze direction and face orientation is also similar to that obtained in previous experiments, but whereas Experiment 1’s Bayesian analysis provided only anecdotal support for that null interaction, in the present experiment the Bayesian analysis suggested substantial evidence for the null. Notably, the effect of gaze direction is consistent with the results of Experiment 1, where the main effect of gaze direction showed faster RTs for direct gaze faces (*M* = 3018.6 ms) than averted gaze faces (*M* = 3436 ms). In the present experiment, the main effect of gaze direction is accompanied by an interaction between exposure duration and gaze direction. Indeed, the single duration at which the effect of gaze direction reaches Bonferroni-corrected statistical significance (3797 ms) is the first that is higher than the RTs found in Experiment 1, confirming a correspondence between the two experiments’ findings.

#### Location response bias

We examined whether participants’ response bias for reporting face location varied across conditions by entering the absolute values of $C{\text{location}}$ scores into a 2 (gaze direction: direct, averted) × 2 (face orientation: upright, inverted) × 7 (exposure durations) repeated-measures ANOVA (Fig. 4b). Response bias significantly decreased with exposure duration $\left(F{(2.57, 71.99)}=4.44,p=.009,\eta{\mathrm{ p}}{2}=0.137\right)$, indicating that as participants’ ability to detect the face increased (shown by higher location d’ scores) they became less likely to exhibit a systematic bias in their preference to report one side or the other. We did not find main effects of gaze direction $\left(F{(1, 28)}=0.149,p=.702,\eta{\mathrm{ p}}{2}=0.005\right)$*,* or face orientation $\left(F{(1, 28)}=0.117,p=.735,\eta{\mathrm{ p}}{2}=0.004\right)$*,* nor any interactions (all p-values > 0.09), suggesting that only exposure duration affected response bias*.* To assess whether the obtained data support the absence of an effect of gaze direction and face orientation, we ran a Bayesian repeated-measures ANOVA to estimate Bayes factors for those null main effects, which indicated substantial evidence for the null hypothesis model of gaze direction $\left(BF{01}=6.899\right)$ and anecdotal evidence for the alternative hypothesis model of face orientation $\left(BF{01}=0.449\right)$.

#### Gaze direction identification sensitivity

We examined whether participants’ sensitivity to gaze direction varied across conditions by entering gaze identification d’ scores—taken over all trials irrespective of location response—into a 2 (face orientation: upright, inverted) × 7 (exposure durations) repeated-measures ANOVA (Fig. 4c). A main effect of exposure duration indicated that sensitivity to gaze direction increased with increasing duration $\left(F{(2.60, 72.77)}=64.25,p<.001,\eta{\mathrm{ p}}{2}=0.696\right)$*.* We also found a main effect of orientation, such that gaze direction identification d’ was significantly higher for upright faces $(M=0.95 \left[1.09\right])$ than for inverted faces $\left(M=0.69 \left[0.93\right]; F{(1, 28)}=34.56,p<.001,\eta{\mathrm{ p}}{2}=0.552\right)$. The interaction between face orientation and exposure duration also reached significance $\left(F{(4.65, 130.24)}=3.67,p=.005,\eta{\mathrm{ p}}{2}=0.116\right)$*.* The advantage in favour of upright faces was evident across most exposure durations, but reached Bonferroni-corrected statistical significance at exposure durations of 1688 $\left(t\left(193\right)= 3.86, p=.014, d=0.717\right)$*,* 2531 $\left(t\left(193\right)= 3.63, p=.033, d=0.675\right)$*,* 3797 $\left(t\left(193\right)= 4.62, p<.001, d=0.857\right)$*,* and 5695 ms $\left(t\left(193\right)= 3.55, p=.044, d=0.660\right)$.

These results add important nuance to the earlier-described effect of gaze direction on location sensitivity, which was not affected by face orientation. Here, our findings show that orientation does affect the ability to explicitly identify gaze direction. This suggests that the processes underlying identification of gaze direction may be distinct from those that allow eye contact to affect breakthrough from CFS, and thus further imply that detection and identification might make independent contributions to responses in b-CFS studies. We return to this in the General Discussion.

It is intriguing that although gaze direction identification sensitivity arises at very short exposure durations, the eye contact effect on location sensitivity (i.e. better detection of direct gaze than averted gaze faces) was only significant at the longer duration of 3797 ms (see “Location sensitivity” results, above). This pattern might be due to the possibility that CFS was ineffective (or less effective) for a subset of our participants. Such participants would have high identification d’ even at low exposure durations, increasing the average d’ scores of the overall sample and causing these scores to depart from zero at shorter durations than they do under effective suppression. Crucially, such participants would not show a direct-gaze advantage on location d’, because if they can see the face well enough to identify its gaze direction, it has already broken through the suppression, obviating an influence of direct gaze on breakthrough and thus eliminating the effect of direct gaze on the ability to detect the face (which is reflected in location d’). This will result in the eye contact effect only becoming apparent at longer exposures, where breakthrough occurs sufficiently often in the overall sample to overcome the null effect introduced by participants for whom CFS is ineffective.

To test this possibility, we ran a follow-up analysis in which we excluded 8 participants who had high gaze direction identification d’ scores at the shortest exposure duration; we repeated the ANOVAs for location d’ and gaze direction identification d’ on the remaining 21 participants (for full details, see the Supplementary Information). The findings are consistent with the possible account described above: Identification d’ rises above zero at 1125 ms for upright faces and 2531 ms for inverted faces; critically, the eye-contact effect on location performance (i.e. higher d’ scores for direct gaze than averted gaze) is now significant at 1125 ms, in addition to the previously-reported 3797 ms (though not in the two intervening durations; we note that in this reduced sample, the data are overall noisier). Therefore, when including only participants for whom CFS was effective, the shortest duration at which we observe an eye contact effect on location d’ is the same as the shortest duration at which there is above-chance identification d’.

#### Gaze direction identification criterion

We examined whether participants’ criterion for reporting direct gaze varied across conditions by entering $C{{\text{gaze identificatio}}}{{{\text{n}}}}$ scores into a 2 (face orientation: upright, inverted) × 7 (exposure durations) repeated-measures ANOVA (Fig. 4d). Here, lower *C* values indicate a more liberal criterion for reporting direct gaze. There was a main effect of face orientation, indicating significantly more liberal criteria for upright $(M=1.05 [1.07])$ than for inverted faces $\left(M=1.13 \left[1.06\right]; F{(1, 28)}=6.78,p=.015,\eta{\mathrm{ p}}{2}=0.195\right)$*.* Unlike in our previous analyses, the main effect of exposure duration was not significant $\left(F{(1.75, 48.95)}=2.53,p=.097, \mathrm{\eta p}{2}=0.083\right)$; and although criteria did become numerically more liberal as exposure duration increased, a Bayesian repeated-measures ANOVA suggested only anecdotal support for the alternative hypothesis of a main effect of exposure duration $\left(BF{01}=0.445\right)$. The interaction between face orientation and exposure duration was also not significant $\left(F{(4.13, 115.63)}=1.86,p=.120, \mathrm{\eta p}{2}=0.062\right)$.

Thus, the key result here is that the criterion for reporting ‘direct gaze’ is more liberal for upright than for inverted faces. As face inversion disrupts configural processing, our findings suggest that such processing may play a role in participants’ criteria for identifying gaze direction.

### Discussion

The second experiment showed that sensitivity to the location of a face stimulus was greater overall for direct-gaze faces than averted-gaze faces, as well as for upright over inverted faces. This is consistent with the findings of previous b-CFS studies^8,9,34,35,63,64^, and suggests that those findings may indeed be due to upright and direct-gaze faces overcoming suppression faster, resulting in greater perceptual sensitivity to them. Also consistent with prior findings, we did not find a significant interaction between gaze direction and face orientation, and a Bayes Factor analysis suggested substantial evidence for the null (i.e. direct gaze enhanced sensitivity to a similar degree for both upright and inverted faces). We return to the interpretation of this point in the General Discussion.

Interestingly, although face orientation did not modulate the effect of gaze direction on *location* sensitivity, we did find that participants’ *identification* of gaze-direction was better for upright than inverted faces. This suggests that the processes underlying detection and identification of the same stimulus are at least partly dissociable, in line with various claims that identification is more dependent upon high-level-processing than detection^41,69–71^.

Importantly, there were no effects of gaze direction on response bias for location. Participants did, however, exhibit a more liberal criterion for reporting direct gaze when the face was upright rather than inverted. Although this result cannot explain the eye-contact effects found in b-CFS studies, it does demonstrate that decision criteria may differ across experimental conditions in studies using perceptual suppression. Generally, therefore, it is important to rule out the possibility that such criterion differences may account for RT effects in standard b-CFS studies.

## General discussion

Do the configural properties of a face and its gaze direction determine how quickly it overcomes interocular suppression? Past studies have claimed that they do, but the methods they used did not employ measures that assess perceptual sensitivity independently of decision criteria. The present study provides a methodologically stringent examination of these two claims about face processing: First, that faces making eye contact overcome suppression from awareness more quickly than faces looking away^9,20,56^. Second, that upright faces overcome suppression faster than inverted faces^9,14,34,35,63,64^. Prior work on these topics has used the b-CFS procedure, which measures how long it takes participants to report that a stimulus has broken through interocular suppression. RTs in this procedure are typically lower for direct-gaze than averted-gaze faces, and for upright than inverted faces. These findings are taken as support for claims of prioritised unconscious processing of direct-gaze and upright faces, allowing them faster access to awareness. We confirmed both findings in Experiment 1, which was a high-powered replication of Stein et al.^9^. However, while such RT differences could reflect enhanced perceptual sensitivity for direct-gaze faces and upright faces, they could also reflect other factors, such as differences in decision criteria and interference between detection and identification processes.

We therefore developed a new variant of the escape-from-suppression paradigm, de-confounding sensitivity and criterion. Participants were presented with CFS-masked faces in trials of predetermined duration, and were then asked to report in which of two possible locations the face had been presented (to measure whether the stimulus had been detected), and whether it was making eye-contact (to measure whether it had been identified). By controlling the amount of visual information available to the participant, and by collecting signal detection measures, our method offers a more robust approach to testing the effects of face orientation and gaze direction on perceptual sensitivity under CFS.

Applying this method, Experiment 2 found that both gaze direction and face orientation can indeed affect participants’ sensitivity to a stimulus. Specifically, participants showed higher sensitivity for detecting the location of faces that were making eye contact, compared to faces whose gaze was averted. Participants also showed higher sensitivity to the location of upright faces, compared to inverted faces. In addition, participants’ identification sensitivity for faces’ gaze direction (whether they were making eye contact or looking away) was greater for upright faces. We also found that participants’ criterion for reporting that a face was making eye contact was more liberal when the face was upright. This latter result is important, because it confirms that properties of the stimuli used in b-CFS experiments (i.e. inverted versus upright faces) can influence decision criteria independently from perceptual sensitivity; standard b-CFS studies cannot dissociate these separate influences on observers’ responses. Although our main focus here is on the effects of gaze, and thus we used a gaze direction identification task, we note that a similar identification task could also be used in further studies for the face orientation factor. Such a task may reveal an orientation identification sensitivity advantage and more liberal criteria for identifying upright, compared to inverted faces, paralleling the present gaze direction findings.

Why do faces making eye contact and upright faces yield better sensitivity under CFS? One potential clue comes from the finding that the eye-contact effect was not disrupted by face inversion, both when measured by RTs (Experiment 1) and by location sensitivity (Experiment 2). Prior work using b-CFS has interpreted the lack of interaction as an indication that gaze processing occurs at the level of low-level features (whose processing is not disrupted by inversion^40,41,43^), prior to configural—or ‘holistic’—face processing^9,20^. However, our finding that inversion does impair gaze identification suggests a more nuanced view. Inversion affects whether a face is seen to have direct gaze but does not affect how direct gaze influences whether a face is seen. This implies that the effect of eye-contact on location sensitivity is not mediated by gaze-detection mechanisms per se, because if it were, we would expect inversion to also affect location sensitivity (as it affects sensitivity for judgments about eye contact). Our results therefore suggest that face *detection* relies on low-level local features (including those that are physically associated with different gaze directions), and is thus not disrupted by inversion, whereas *identification* of gaze-direction depends on configural processing that is disrupted by inversion.

Importantly, even if the eye-contact effect demonstrated here relies predominantly on low-level processing, this does not mean that it is irrelevant to the processing of eye contact in daily life. These low-level cues may draw attention to a stimulus, and enhance its processing, even if they do not reflect specific computations for the detection of eye contact. Furthermore, processing these low-level cues may be required for more complex social cognition involving eye contact, such as joint attention. Future work will need to clarify the relation between the eye-contact effect and more complex but related social cognitive functions. This may be especially relevant for studies with clinical populations. For example, b-CFS studies have reported that the eye-contact effect is preserved in schizophrenia^55^ but impaired in autism^28^; it is unclear whether this reflects differences in high-level processing, or differences in low-level processing that have emerged from a lifetime of divergent perceptual learning. Such considerations may also be important when considering a range of other claims concerning perceptual advantages related to socially-relevant features that are assumed to require high-level perceptual integration^72–74^ (but see^75^).

Non-speeded tasks in which the exposure to a stimulus of interest is controlled by the experimenter, like in our Experiment 2, have the advantage of allowing direct measurement of perceptual sensitivity, the ability to discriminate a signal from noise in a given amount of sensory information. By combining detection and identification tasks, we were able to test how location sensitivity and gaze direction identification sensitivity arise as visual exposure increases. Although the overall increase in sensitivity with exposure duration followed similar trajectories for the two tasks, the differences between them (e.g. the influence of inversion on identification but not detection, discussed above), suggests that participants might accumulate visual evidence differently for detection and identification of facial features.

Further investigation of this possibility may benefit from the use of speeded-response paradigms: For instance, like our adaptation of the method of constant stimuli, the response signal (or cued-response speed-accuracy trade-off) procedure^76–79^ employs a range of fixed exposure durations or fixed stimulus-cue time lags; however, this paradigm involves a speeded response—following each pre-determined stimulus duration (or stimulus–response lag), participants are cued to provide their response as fast as they can within a very brief (typically around 300 ms) response window. Even for stimuli that are clearly visible, very short exposures (or stimulus-cue lags) lead to a high error rate as there is not enough time to process the stimulus, associate it with the appropriate response, and then plan and execute that response; the paradigm thus uses a speed-accuracy trade-off to assess the speed of perceptual-motor decision making, and could be useful in assessing how these processes play out for detection and identification of stimuli under CFS.

A different approach, which could be applied fruitfully to elucidating the perceptual decision-making mechanisms of detection and identification tasks, is drift diffusion modelling (DDM^80,81^). DDM explains behaviour in two-choice discrimination tasks by fitting a model to RT data; the model estimates parameters denoting drift rate (the speed of evidence accumulation) and decision boundaries (the amount of information required for a decision) for each of the possible responses, as well as initial bias and the duration of processes that are not part of the decision making (e.g. execution of motor responses). Although the number of trials in our exact replication of Stein et al. (2011)^9^ in Experiment 1 did not provide sufficient power for applying such model fitting, this approach has been successfully applied to the RT data of b-CFS (e.g. in the context of emotional processing; McFadyen et al.^82^). In the present context, DDM could potentially help to extend our findings by using both detection and identification tasks in a b-CFS paradigm, and examining whether differences between the models’ estimates for the different tasks suggest differences in evidence accumulation speed, bias, or decision boundaries.

Our measures of perceptual sensitivity did not incorporate an explicit assessment of perceptual awareness: Although it is reasonable to assume that increased sensitivity arises as stimuli break into awareness, it remains possible that differences between conditions’ d’ scores at a given duration may reflect contributions from unconscious processes that could influence perceptual reports (as occurs in blindsight^83^, and has been shown under masking^84^), in addition to differences in conscious perception at the specific exposure duration. A remaining challenge for further research, therefore, is to ascertain the relative contribution of conscious and unconscious processes to psychophysical sensitivity. This could be accomplished by adding specific measures of awareness, such as psychophysical assessments of metacognitive sensitivity based on either confidence ratings or the perceptual awareness scale^65,85,86^ to either the detection, identification, or both tasks. We note that this is technically challenging, as each additional measure adds to the number of responses required on each trial, and to the overall number of trials required for robust psychophysical measures at each duration^87^); the present findings could therefore inform such follow-ups by suggesting a restricted set of durations for such an assessment.

In summary, by using a stringent psychophysical procedure to study the factors that affect how faces overcome perceptual suppression, we have demonstrated that the configural features of a face and its gaze direction influence perceptual sensitivity, an effect that may be mediated by unconscious processing. This new procedure addresses the limitations of the b-CFS procedure, the most popular method used for this purpose in recent years. Using our method, we found robust evidence for two effects that had been reported with b-CFS: an advantage in detection of direct-gaze over averted-gaze faces (eye-contact effect) and of upright over inverted faces (face-inversion effect); but unlike with b-CFS, we found these effects by measuring sensitivity directly, controlling for response bias and identification criterion differences. Furthermore, we demonstrated—in a way that is not possible with b-CFS—that criterion differences for reporting features of stimuli that are presented under interocular suppression can arise between different stimulus conditions independently of sensitivity (as found here for the effect of face orientation on gaze identification criteria). Critically, the fact that our findings confirm previously-reported effects does not mean that all b-CFS results are reliable; on the contrary—it means that all b-CFS findings (including, and especially, the ones that have not failed replication attempts) should be submitted to rigorous methods to establish whether they are attributable to effects on perceptual sensitivity, decision criterion, or both.

## Supplementary Information

Supplementary Information.
