# Supplementary material for: Gaze direction and face orientation modulate perceptual sensitivity to faces under interocular suppression
Source: Sci Rep. 2022 May 10;12:7640. doi: 10.1038/s41598-022-11717-4 (PMC9090921; doi:10.1038/s41598-022-11717-4)

SUPPLEMENTARY INFORMATION

Gaze direction and face orientation modulate perceptual sensitivity to faces under interocular suppression

Renzo C. Lanfranco, Timo Stein, Hugh Rabagliati, & David Carmel

Supplementary Analysis for Experiment 2: Excluding participants with ineffective suppression at short exposure durations

In Experiment 2, above-chance gaze direction identification sensitivity was evident at shorter exposure durations than those in which there was an eye-contact effect on location sensitivity. As explained in the main text, this may be due to the presence of participants for whom CFS was ineffective (or not fully effective). To test this possibility, we ran a follow-up analysis in which we excluded participants (n = 8) who had high gaze direction identification sensitivity (d′ > 0.3) at the shortest exposure duration (500 ms); we then repeated the ANOVAs for location d′ and gaze direction identification d′ on the remaining participants (n = 21).

Gaze direction identification sensitivity

We entered gaze direction identification d′ scores into a 2 (gaze: direct, averted) × 7 (exposure durations) repeated measures ANOVA (Figure S1a). As in the main analysis, we found a main effect of exposure duration, indicating that sensitivity to gaze direction increased with increasing duration ($F_{(2.66,\,53.16)} = 53.247, p < .001, \eta p^2 = .727$); a main effect of face orientation, demonstrating higher identification d′ for upright faces ($M = 0.711\ [0.784]$) than for inverted faces ($M = 0.485\ [0.636]$) ($F_{(1,\,20)} = 24.272, p < .001, \eta p^2 = .548$); and a significant interaction between these factors ($F_{(6,\,120)} = 3.304, p = .005, \eta p^2 = .142$). Pairwise comparisons revealed an advantage for upright over inverted faces at 3797 ($t(20) = 3.384, p = .044, d = 0.706$) and 5695 ms of exposure ($t(20) = 3.338, p = .049, d = 0.697$).

To establish the shortest duration at which gaze direction identification d′ is significantly higher than zero, we ran a series of uncorrected one-sample t-tests against zero, to find at which exposure duration identification sensitivity was above chance. We found that identification d′ was significantly above zero by 1125 ms of exposure for upright faces ($t(20) = 2.597$, $p = .017$, $d = 0.567$), and by 2531 ms for inverted faces ($t(20) = 3.212$, $p = .004$, $d = 0.701$); note that identification d′ was marginally above chance by 1688 ms for inverted faces ($t(20) = 1.865$, $p = .077$, $d = 0.407$).

Location sensitivity

We entered location d′ scores into a 2 (gaze: direct, averted) × 2 (face orientation: upright, inverted) × 7 (exposure durations) repeated measures ANOVA (Figure S1b). As in the main analysis, we found a main effect of exposure duration, indicating that sensitivity increased

with exposure duration ($F_{(2.47,\ 49.34)} = 128.988, p < .001, \eta p^2 = .866$); a main effect of gaze direction, confirming that participants were more sensitive to the location of direct-gaze faces ($M = 1.102$ [0.869]) than averted-gaze faces ($M = 0.988$ [0.857]), ($F_{(1,\ 20)} = 20.09, p < .001, \eta p^2 = .501$); and a main effect of face orientation, indicating a sensitivity advantage for upright faces ($M = 1.126$ [0.892]) over inverted faces ($M = 0.966$ [0.829]) ($F_{(1,\ 20)} = 7.63, p = .012, \eta p^2 = .276$). The interactions between exposure duration and gaze direction ($F_{(6,\ 120)} = 10.889, p < .001, \eta p^2 = .353$), and between exposure duration and face orientation ($F_{(6,\ 120)} = 3.679, p = .002, \eta p^2 = .155$) reached significance. No other interaction reached significance. The eye-contact effect (i.e. advantage of direct-gaze over averted-gaze faces) arose at 1125 ($t(20) = 3.976, p = .01, d = 0.422$) and 3797 ms ($t(20) = 7.466, p < .001, d = 0.793$) of exposure. The face-inversion effect (i.e. advantage of upright over inverted faces) arose at 1688 ($t(20) = 3.374, p = .032, d = 0.481$), 2531 ($t(20) = 3.329, p = .036, d = 0.474$), and 3797 ms of exposure ($t(20) = 3.228, p = .045, d = 0.46$).

Figure S1. Experiment 2 results excluding participants for whom CFS was ineffective (i.e. identification d' > 0.3 with 500 ms of exposure). (a) Gaze direction identification sensitivity: Identification d' increased with exposure duration, and a main effect indicated it was significantly higher for upright faces. d' was significantly above zero for upright faces by 1125 ms and for inverted faces by 2531 ms. (b) Location sensitivity: d' increased with exposure duration. Main effects indicated a significant advantage for direct-gaze over averted-gaze faces and a significant advantage for upright over inverted faces. Daggers indicate statistically significant differences between gaze directions. Asterisks indicate statistically significant differences between face orientations. Error bars represent 95% CI.

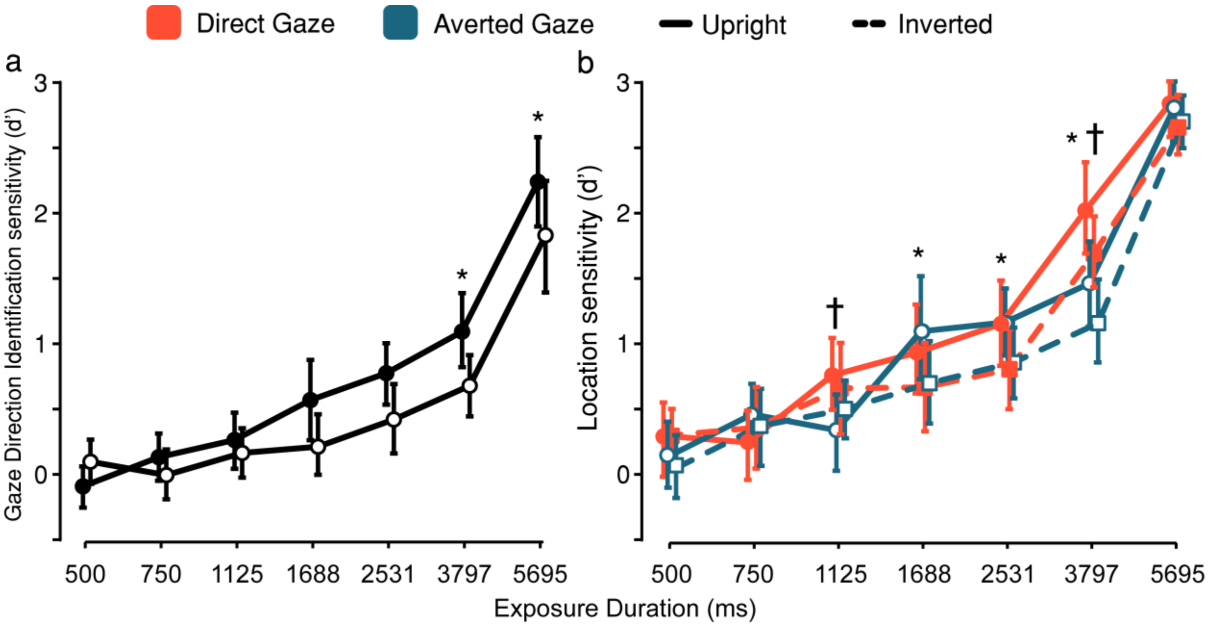

Supplement: Supplementary file 1 — Supplementary Information. [file 41598_2022_11717_MOESM1_ESM.pdf]
